# Friends with Benefits: Chemokines, Glioblastoma-Associated Microglia/Macrophages, and Tumor Microenvironment

**DOI:** 10.3390/ijms23052509

**Published:** 2022-02-24

**Authors:** Elena Codrici, Ionela-Daniela Popescu, Cristiana Tanase, Ana-Maria Enciu

**Affiliations:** 1 Victor Babes National Institute of Pathology, 050096 Bucharest, Romania; cristianatp@yahoo.com; 2 Department of Clinical Biochemistry, Faculty of Medicine, Titu Maiorescu University, 031593 Bucharest, Romania; 3 Department of Cell Biology and Histology, Carol Davila University of Medicine and Pharmacy, 050474 Bucharest, Romania

**Keywords:** tumor microenvironment, glioblastoma, glioblastoma-associated microglia, glioblastoma-associated macrophages, myeloid-derived suppressor cells, dendritic cells, tumor infiltrating lymphocytes, soluble factors, chemokine, specific receptors, gene editing

## Abstract

Glioma is the most common primary intracranial tumor and has the greatest prevalence of all brain tumors. Treatment resistance and tumor recurrence in GBM are mostly explained by considerable alterations within the tumor microenvironment, as well as extraordinary cellular and molecular heterogeneity. Soluble factors, extracellular matrix components, tissue-resident cell types, resident or newly recruited immune cells together make up the GBM microenvironment. Regardless of many immune cells, a profound state of tumor immunosuppression is supported and developed, posing a considerable hurdle to cancer cells’ immune-mediated destruction. Several studies have suggested that various GBM subtypes present different modifications in their microenvironment, although the importance of the microenvironment in treatment response has yet to be determined. Understanding the microenvironment and how it changes after therapies is critical because it can influence the remaining invasive GSCs and lead to recurrence. This review article sheds light on the various components of the GBM microenvironment and their roles in tumoral development, as well as immune-related biological processes that support the interconnection/interrelationship between different cell types. Also, we summarize the current understanding of the modulation of soluble factors and highlight the dysregulated inflammatory chemokine/specific receptors cascades/networks and their significance in tumorigenesis, cancer-related inflammation, and metastasis.

## 1. Introduction

Glioma is the most common primary intracranial tumor and has the greatest prevalence of all brain tumors (about 46% of all intracranial tumors). Among them, glioblastoma (GBM), a WHO grade IV glioma, is the most frequent and aggressive primary malignant brain tumor, being described by extensive infiltration of resident immune cells (microglia) and new recruited immune cells (peripheral macrophages) in the tumor, as well as ubiquitous infiltration of tumor cells in the healthy tissues around the tumor [1]. New in-depth studies/investigations are needed to identify effective therapy methods for GBM due to its high malignancy and poor prognosis caused by the GBM heterogeneity and the complexity of the microenvironment within a tumor [2].

Soluble mediators (e.g., chemokines, cytokines), extracellular matrix components (e.g., collagen, glycoproteins), tissue-resident cell types (e.g., neurons, astrocytes, endothelial cells, pericytes, and fibroblasts), as well as resident (e.g., microglia) or new recruited (e.g., bone marrow-derived macrophages) immune cells, comprise the GBM microenvironment. Despite the abundance of immune cells, a profound state of tumor immunosuppression is supported and developed, posing a considerable barrier to immune-mediated elimination of cancer cells. Numerous researches suggest that different GBM subtypes present differences in their microenvironment, but the relevance of the microenvironment in treatment response is unknown yet [3].

In the last years, understanding the biology of gliomas has progressed significantly, including the discovery of glioma stem cells (GSCs), which are thought to be responsible for tumor recurrence. Understanding the microenvironment and how it changes after various therapies is critical because it can influence the remaining invasive GSCs and lead to recurrence. Current researches emphasize the relevance not only of cancer-infiltrating immune cells but also circulating immune cells as indicators of recurrence and poor patient outcomes [4,5,6,7].

Tumors develop in close interaction with their microenvironment, which includes a continual struggle between the growing tumor and the host immune system. It was observed that enhancing a tumor’s immune response in the right way can result in long-term therapeutic responses and patient benefit. Understanding the role of the immune contexture and molecular subtypes in various tumors is a major knowledge gap with limited sagacity to drive rational immunotherapy combinations. Numerous studies have confirmed that patients with glioblastoma experience both systemic immunosuppression, characterized by impaired cell-mediated immune function, that occurred after radiation or chemotherapy and local immunosuppression, which occurred in the tumor microenvironment (TME) [8]. Gliomas have been named “cold tumors” in the literature due to their high immunosuppressive TME, where “cold tumors” are defined by the absence of T-cell infiltrate within the TME and are less responsive to immunotherapies [9].

This review aims to describe the key roles of tumor microenvironment in the GBM development, as well as immune-related biological processes, which underpin the intercellular communication between different cells types (in the first part of the review, Section 2—Tumor microenvironment), modulation of soluble factors, respectively dysregulated inflammatory cascade of chemokines (in the second part of the review, Section 3—Dysregulated inflammatory cascade of chemokines and their receptors), and therapy (in the third part of the review, Section 4—Cell therapy).

## 2. Tumor Microenvironment—Regulator of Cancer Progression

**Figure 1:**
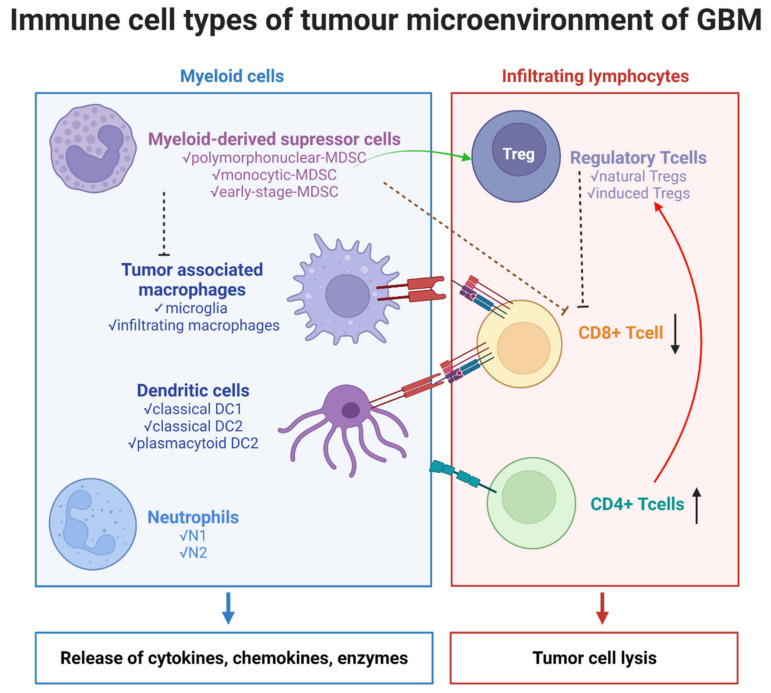
Immune cells types of tumor microenvironment of GBM. The illustration was created with Bioreder.com.

Treatment resistance and tumor recurrence in GBM are mostly explained by considerable alterations within the TME, as well as extraordinary cellular and molecular heterogeneity [10]. The glioma microenvironment contains a large variety of cell types (Figure 1), such as:

(a) immune cells:
  ✔ myeloid cells: tumor-associated macrophages (TAMs)—resident microglia and bone marrow-derived macrophages (BMDMs), dendritic cells (DCs), neutrophils/tumor-associated neutrophils (TANs), myeloid-derived suppressor cells (MDSCs);
  ✔ lymphoid cells—T cells: CD8+ cytotoxic T cells, CD4+ helper T (Th) cells, and regulatory T cells (Tregs);
(b) non-immune cells: tissue-resident cells—neurons and astrocytes, endothelial cells, and pericytes.
(c) tumor cells, glioma stem cells (GSCs).

All cells are embedded in an extra-cellular matrix (ECM) of fibrous proteins and glycolproteins, which provide support [11,12,13,14,15,16]. In the early stages of cancer, the host defense, which consists of both innate and adaptive immune cells, is involved in immune surveillance. Still, during tumor development, certain tumor cells can evade immune monitoring, allowing them to attract and recruit immune cells and shift their original role to one of the tumor’s accomplices [14,17].

GBM has been characterized as an immunosuppressive tumor that can alter the immune system, respectively increase the tumor immune escaping, by different mechanisms:

(a) secretion of several immunosuppressive factors, like cytokine, chemokines, growth factors (IL-6, IL-10, IL-1, TGF-β, prostaglandin E2, bFGF) by tumor cells, microglia, and tumor-associated macrophages (TAMs) [13]
(b) expression of some immunosuppressive cell-surface factors, like CD95, CD70, programed cell death protein-1 ligand (PD-L1), CTLA-4, and gangliosides [18,19]
(c) recruiting of immunosuppressive inflammatory cells to the TME [8,20].

The concurrent result of these mechanisms leads to suppressing of NK activity and T-cell activation and proliferation, inducing T-cell apoptosis, down-regulating the MHC class II expression, and switching TAMs to an M2 immunosuppressive phenotype [21,22]. Understanding all the above-mentioned mechanisms of immune escape are essential to achieve the GBM immunotherapy, respectively, to treat the tumor by overcoming the tumor cells’ immunological resistance.

### 2.1. Myeloid Cells in Glioma Microenvironment

#### 2.1.1. Tumor Associated Macrophages (TAMs)

TAMs are one of the most prevalent populations in the tumor stroma, according to meta-analysis research, and their abundance appears to be associated with poor overall survival in patients with head and neck cancer, gastric cancer, and urogenital cancer [23]. In GBM tissue, TAMs account for about one-third of TME’s cells, and the number of TAMs is related directly to tumor grade and inversely to patients’ survival [15,24,25].

TAMs are a heterogeneous population based on their origin, localization within the tumor, and their functions [3,26].

In brain tumors, TAMs could have different origins: either newly recruited cells with a peripheral origin, from bone marrow-derived macrophages (BMDMs) named glioblastoma-associated macrophages, or are represented by tissue-resident macrophages/brain-intrinsic microglia named glioblastoma-associated microglia. According to their localization, BMDMs can be subdivided into meningeal macrophages, choroid plexus macrophages, and perivascular macrophages [3,27,28,29]. Also, a different localization in the perivascular area of the tumor was observed [30]; their localization in the tumor appears to depend on their phenotypes [31]. Thus, while resident microglia were localized to peritumoral regions, BMDMs were localized especially to the perivascular niche [14].

Initially, a binary M1/M2 functional polarization of TAMs exist: a pro-inflammatory M1 state, characterized by the classical activation of immune receptors TLR2/4 and the production of pro-inflammatory cytokines (TNF, IL-1β), and the anti-inflammatory M2 state, with the production of ARG1, IL-10, and IL-4 [32]. However, because macrophages in tissue are exceedingly varied, with dynamic and variable phenotypes and activities that are constantly affected by their tissue milieu, this categorization is too basic to describe the phenotype and functions of TAMs in malignancies [33,34]. In addition, a link between tissue hypoxia, a hallmark of GBM, and TAMs polarization was discovered. M1 macrophages are presented within normoxic tumor regions, while M2 macrophages are presented within hypoxic regions [14,35]. In light of these findings, TAMs polarization is dependent on multiple different signals existing in the microenvironment [36]. CD163, CD204, Arg1, Mrc1, Chi3l3, Socs2, Fizz-1, and Ccl2 mRNA expression in M2 macrophages, Nos2, IL12b, and Ciita expression in M1 macrophages, were suggested as markers to discriminate between M1- and M2-like states [37].

##### Glioblastoma-Associated Microglia—Resident Cells

Microglia are the resident mononuclear macrophages of the CNS, constitute ~5–20% of the total glial cells population in the parenchyma and plays key roles in immune surveillance, preserves neuroimmune homeostasis, supporting the neural networks development and survival of neuronal precursor cells, contributing to the elimination of the apoptotic cells [38,39,40,41]. When microglia are confronted with a challenge, such as tumor development, their immune response might be severely hampered or maladapted [42].

Microglia were formerly assumed to be solely formed in the Yolk sac during embryogenesis from erythro-myeloid progenitor cells (EMPs) [43]. Recent research has found that 25% of microglia in the brains of P2-P24 mice are descended from hematopoietic-monocytic cells, using Ccr2-CreER for lineage tracing [44,45]. Besides, new studies have discovered microglial transcriptome heterogeneity in different mouse brain areas, indicating the existence of many microglial phenotypes based on topological distribution and protein expression levels in the human brain [46]. Moreover, it has been demonstrated that the spatial distribution of human microglia impacts their transcriptional conditions, which fluctuate with age and the pathophysiology of brain tumors [47,48].

When microglia are in surveillance mode, they have a highly ramified shape, but when triggered, they transition rapidly to an amoeboid morphology [42]. In tumor-associated microglia, metabolism and pro-inflammatory cytokine-related genes are overrepresented [49].

##### Glioblastoma-Associated Macrophages—Newly Recruited Cells

The brain-infiltrating bone marrow-derived macrophages (BMDMs) originate from hematopoietic stem cells [50]. TAMs mediate tumor recurrence in mice, according to in vivo research of GBM [51,52]. TAMs, which support glioma proliferation and regrowth, developed a recurrence-specific phenotype response to radiation. By pointing of TAMs with CSF-1R inhibition combined with radiotherapy blocked the acquisition of pro-tumorigenic phenotype and substantially improved survival in preclinical models [34,53].

One of the major obstacles in understanding the functional role of these two ontogenetically separate myeloid cell populations with comparable immune regulatory traits is the segregation of microglia and BMDMs, which may be achieved using specific biomarkers [54,55,56]. These two different cell populations share positivity for CD11b, CD68, CX3CR1, F4/80, and ionized calcium-binding adapter molecule 1 (IBA1) [57], but by new techniques, like transcriptional profiling and in vivo two-photon (2P) microscopy, obvious molecular differences between these two groups were revealed [49,58,59].

Conventionally, microglia are defined as CD11b^+^/CD45^low^ and CX3CR1^+^/CCR2^low^, whereas BMDMs population is defined as CD11b^+^/CD45^high^ and CX3CR1^low^/CCR2^+^. Microglia, an inflammatory condition, can express CD45, becoming hardly distinguishable from BMDMs [18,60].

Recently, new specific biomarkers (Table 1) have been suggested.

With increasing malignancy grade, P2RY12 expression in microglia changes from cytoplasmic to nuclear, and nuclear expression of P2RY12 coincides with that of the M2 markers CD163 and CD204 in high-grade malignancies. According to this observation, P2RY12 positive microglial cells in high-grade gliomas have taken on an amoeboid character [64].

Crosstalk between TAMs and glioma cells creates a tight dependency that increases TAM recruitment and tumor development and invasion. Thus, TAMs secreted a plethora of molecules that promote tumor development and invasion by acting on glioma cells and GSCs in the perivascular niche:

✔ TGF-β—induces secretion of matrix metalloproteinase 9 (MMP9), disruption of ECM, enhances GSCs invasion [65].
✔ TGF-β2—induces secretion of MMP-2, inhibited the tissue-inhibitor metalloproteinases 2 (TIMP-2), stimulating tumor invasion [66].
✔ IL-1β—induces p38 MAPK pathway activation, CCL2/MCP-1 production, stimulates GSC proliferation [16].
✔ Stress-inducible protein 1 (STI1) and EGF promotes GBM growth and invasion [67,68].
✔ PDGFRB—stimulates glioma-cells migratory capacity and tumor development [69].

On the other hand, glioma-cells release chemoattractant molecules that recruit TAMs and promote tumor growth: CCL2 [49], CCL7 (or MCP-3) [70], IL-33 [71], GDNF (Glial cell line-derived neurotrophic factor) [72], MCP-1 (CCL2), SDF-1 (CXCL12), CSF-1 (M-CSF), GM-CSF, and EGF [11].

The binding of immunogenic antigens to microglia/macrophages, through different surface receptors, such as toll-like receptors (TLRs), nucleotide-binding oligomerization domain-(NOD)-like receptors, and scavenger receptors (SRs), leads to their activation towards a pro-inflammatory phenotype, as follows:

- the activation of transcription of STAT1, NF-Kb, TNF-a, increases the pro-inflammatory chemokines and cytokines secretion—(IL)-1α, -1β, -6, -12, -23, CCL2–5 and CCL8–11.
- the activation of redox molecules: NADPH oxidase, inducible nitric oxide synthase (iNOS) for nitric oxide production.

M2 polarized macrophages are further divided into three types: M2a, M2b, and M2c phenotypes, and express general (CD68, CD11b, and IBA1) and specific (CD80^low^/CD86 ^low^, CD163, CD204 and CD206) markers [36,73].

Bidirectional communication between tumor cells and a subset of M2-polarized BDBM—M2c cells was mentioned. GBM cells release soluble factors, like IL10, TGF-beta, and glucocorticoids, which stimulate the growth of the M2c population, which in turn stimulates tumor cells proliferation. As a result, GBM’s TAMs mostly acquire M2 polarization, and an unbalanced M1-M2 interchange may be distinguished [74,75,76]. As evidenced by oncolytic virotherapy’s ability to restore M2 to M1 phenotype, immunosuppressive pro-tumoral M2 macrophages are a viable therapeutic target [57], which will be briefly presented in Section 4 of the review.

#### 2.1.2. Myeloid-Derived Suppressor Cells (MDSCs)

MDSCs are a heterogeneous cellular population and a major constituent of the suppressive network that promotes tumor development and contributes to therapeutic resistance. Three major types of MDSCs were found: granulocytic or polymorphonuclear MDSCs (PMN-MDSCs), monocytic MDSCs (M-MDSCs) and early-stage MDSCs (eMDSCs) [77]. Although PMN-MDSCs and M-MDSCs are phenotypically and morphologically comparable to neutrophils and monocytes, all three categories of MDSCs have distinct biochemical and genetic properties, including the ability to block immune responses [78]. In cancer patients, MDSCs are cells that co-express the following myeloid differentiation markers: CD11b and CD33 are present, but MHC class II molecules and HLA-DR indicators are absent [79]. In humans, M-MDSCs are described as CD14^+^CD11b^+^CD33^+^HLA-DR^low^/−CD15^−^, PMN-MDSCs as CD14^−^CD11b^+^CD33^+^HLA-DR^low^/−CD15^+^ (or CD66+), and eMDSCs as Lin− (i.e., CD3^−^, CD14^−^, CD15^−^, CD19^−^, CD56^−^, HLA-DR^−^, and CD33^+^ [77].

MDSCs decrease immune responses by reducing cytotoxic T cell antitumor activity [80], suppressing NK cells [81], suppressing macrophage and dendritic cell function [82], and also inducing Tregs (regulatory T cells) and Bregs (regulatory B cells) [83]. In MDSCs exposed to hypoxia, there was distinguished an up-regulation of CD45 tyrosine phosphatase activity and a down-regulation of STAT3 transcription factor activity, a fact which facilitated the differentiation of M-MDSCs into TAMs [84].

Alban T.J. et al., showed that MDSC levels in GBM patients with long survival were similar to those in low-grade glioma patients, whereas superior MDSC accumulation at the time of recurrence indicates a worse prognosis in patients with GBM [85]. This working group continued research and found that the CD74 receptor might have an important role in GBM MDSC biology because, in the tumor microenvironment, the most commonly detected MDSCs were M-MDSCs, which highly express CD74 as a MIF receptor [86]. Several additional investigations have mentioned that the intratumoral and systemic blood frequency of MDSCs increases with glioma development and correlates with grade of malignancy and prognosis in glioma patients [87,88,89,90]. Also, all subsets of MDSC were found in the blood and tumors of GBM patients, with a vast majority for PMN-MDSCs in patients’ blood [87].

Gielen et al., found that CD14-positive M-MDSC subpopulation was significantly increased in peripheral blood from patients with GBM, while CD15-positive PMN-MDSC subpopulation was found to be elevated almost entirely in tumor tissue [89]. On the other hand, a different study discovered that distinct MDSC subpopulations were observed in GBM patients, and a higher PMN-MDSCs density in peripheral blood and also at the tumor site have a key role in GBM-induced T-cell suppression [88].

Starting with the information that is currently available, respectively MDSCs numbers elevated in the circulation of GBM patients and reversible T cell dysfunction at that level, Raychaudhuri B. et al., used sunitinib, a tyrosine kinase inhibitor, to treat mouse brain tumor models and reported an overall decrease in granulocytic and monocytic MDSCs, enhanced T cell proliferation, and IFNγ production, all of which are linked to tumor shrinkage and better survival. Moreover, they showed that the majority of MDSCs population (CD33+HLADR-) were eMDSCs with negative lineage (CD14-CD15-) followed by PMN-MDSCs population (CD15+CD14-) and M-MDSCs population (CD15-CD14+) subtypes [91].

Recently, in a mouse model of glioblastoma, it was revealed that MDSCs are distributed in a gender specific manner. M-MDSCs were enhanced in the male tumors, while PMN-MDSCs were higher in the blood of females, and reduction of PMN-MDSCs prolonged survival only in female mice. Furthermore, proliferating M-MDSCs was shown to be more common in male malignancies, and a high PMN-MDSC/IL1 gene signature was linked to a poor prognosis in females, suggesting that MDSC subsets differentially drive immune suppression in a gender specific manner and might be leveraged for therapeutic intervention in GBM [92].

The discovery of new specific biomarkers that discriminate between different types of MDSCs and separate MDSCs from normal monocytes or neutrophils will help to further distinguish these populations, elucidate their activities, and understand the mechanisms behind their accumulation in GBM patients. In this regard, a recent study that uses the lectin-type oxidized LDL receptor 1 (LOX-1) marker, which has emerged as a particular marker of human PMN-MDSCs, has begun to shed light on the involvement of neutrophils and PMN-MDSCs in patients with GBM [93]. Furthermore, Chai E. et al., demonstrated that the LOX-1+ (lectin-type oxidized LDL receptor 1) PMN-MDSC decreased T-cell proliferation, resulting in increased immunological suppression, which may play a role in the development of GBM. The prevalence of LOX1+PMNs in the peripheral blood and tissue of GBM patients, but not LOX1-PMNs, was shown to be inversely associated with the amount of effector immune cells in GBM patients, and was linked to early recurrence and disease progression. LOX1+PMNs had a PMN-MDSC profile, with elevated levels of reactive oxygen species (ROS), ARG1, and iNOS, as well as the capacity to limit CD3+ T cell proliferation in an ARG1/iNOS-dependent manner [94].

Altogether, the studies mentioned above point out those different MDSC sub-populations that can co-exist in the blood and tissue of GBM patients, having different roles and distribution [34]. However, it is uncertain if a specific subgroup of MDSCs predominates in human gliomas, and the role of MDSCs in GBM recurrence is not well defined, thus more research is needed to elucidate this important aspect.

#### 2.1.3. Neutrophils in GBM

Neutrophils (PMNs) are predominant circulating leukocyte populations in humans and represent one of the first defense mechanisms in the host organism. When altered, they can contribute to chronic inflammation and carcinogenesis in different malignancies. In the last years, neutrophils are becoming more widely recognized as an important component of the TME, attracted by molecules like IL-8, TNFa, and CCL2, released by malignant or surrounding cells [95]. Different subgroups of neutrophils can coexist in the same cancer patient. This heterogeneity depends on the cancer type, tumor growth, and neutrophil maturation stage [96,97].

Tumor-associated neutrophils (TANs) are a heterogeneous population, divided into two subpopulations based on their polarization: N1 phenotype (pro-inflammatory and anti-tumorigenic) and N2 phenotype (tumorigenic) [98]. The TGFb signaling promotes N2 phenotype (tumorigenic), whereas the IFNb signals or the inhibition of TGFb signaling facilitated N1 phenotype (anti-tumorigenic) [99]. Also, while N1 neutrophils are involved in tumor cell cytotoxicity, activation of T cells, and prevention of carcinogenesis, N2 neutrophils stimulate cancer development and invasion, angiogenesis, and immune suppression [95]. The activity of the N1 type of TANs is decreased as a tumor progresses and predominantly shifts to the N2 phenotype [100,101].

In glioblastoma multiforme patients, neutrophils can penetrate the brain-blood barrier (BBB) and the brain–tumor barrier (BTB) and enter the tumor [102]. A high number of neutrophil infiltration and their activation are related to poor prognosis for GBM patients [103,104,105,106].

Increased neutrophil degranulation has been connected to an increase in arginase1 (Arg-1) levels, which has been linked to immunological suppression in GBM patients. These factors together limit T-cell proliferation in an ARG1-dependent manner [107]. Recent studies suggest that tumor injuries early in cancer progression may facilitate the recruitment of neutrophils to the tumor site, and that neutrophils and ferroptosis are linked to necrosis and predict poor survival in GBM patients via a mechanism involving iron-dependent lipid peroxide accumulation within the tumor [93,108].

Fossati G. et al., observed that in high-grade gliomas, the TAN infiltration rate is high compared to low-grade gliomas [102]. Consecutively, other studies have discovered that the quantity of tumor infiltrating neutrophils corresponds with glioma grade and is a negative prognostic factor for resistant patients [109,110]. Neutrophils are frequently discovered in the tumor core of GBM patients [111]. Neutrophils infiltrate the tumor site due to IL8, MIF, and CXCL8 secretion [112,113]. CXCL8 also recruits chemotactic neutrophils to tumor sites and stimulates them to produce a variety of chemo-attractant molecules [114,115].

Neutrophils might generate neutrophil extracellular traps (NETs), which shield glioma cells in the brain and promote the growth of both primary and metastatic cancers [116]. Neutrophils/NETs produce proteins such as elastase within the TME, which helps neutrophil and glioma cancer cells infiltrate at the tumor site [117]. Furthermore, by directly promoting GBM-initiating cells’ proliferation and migration through the up-regulation of S100A4, these cells induced the transition to a mesenchymal phenotype, favoring cancer invasion and resistance to anti-VEGF therapies [118].

Rahbar A. et al., demonstrated that, in GBM patients, an enhanced neutrophil activity was correlated with high levels of interleukin IL-12p70 and a shorter time to tumor progression, than in patients with decreased or without neutrophil activity. Thus, the neutrophil activation was found to be an early indicator of GBM progression [110].

In an in vivo model was demonstrated that mutant IDH1 glioma, which is less aggressive than the wild-type IDH1 glioma, has low tumor TAN infiltration, and it is associated with the down-regulation of genes involved in chemotaxis. Thus, reduced immune infiltrates in mutant IDH1 glioma could play a role in the aggressiveness differences between mutant and wild-type gliomas [119].

In human glioma cells, bidirectional cross-talk between tumor cells and neutrophils via the Fas (APO-1, CD95)/FasL pathway increased cellular viability and stimulated neutrophil cytokine production (IL-6, IL-8) [112].

The complex mechanism of neutrophil recruitment and the interaction of these cells with other TME cells in GBM development is unclear [33].

Once the inflammation has subsided, macrophages phagocytose the neutrophils. Furthermore, the death of recruited neutrophils triggers an immunosuppressive cascade, similar to that triggered by type M2 macrophages in the tissue healing process [120].

To sum up, neutrophils and PMN-MDSCs may coexist in GBM, suggesting that their activities in brain tumors are distinct. Understanding their phenotypic and functional variability would help researchers better comprehend their role in immunological and therapeutic responses to GBM, as well as their clinical significance [34].

To summarize, the findings mentioned above suggest that inflammatory mediators, such as TAMs, TANs, and related signaling pathways, have a tumorigenic role in glioma.

#### 2.1.4. Dendritic Cells in GBM

Dendritic cells (DCs) are a heterogenous group of antigen-presenting cells (APC) that play an important role in the initiation and regulation of innate and adaptive immune response through capturing, processing, and presenting the antigen to T and B cells [121].

DCs are classified into three groups, according to their immunophenotypic profile: classical DCs (cDC) and plasmacytoid DCs (pDC), which are present and active under steady-state conditions and monocyte-derived DCs (moDC), which arise only during inflammation [122]. Two distinct subsets of cDCs were described: cDC1s and cDC2s [123]. Human cDC1s express CD11c^low^, HLA-DR+, XCR1+, CLEC9A+, DNGR1, and CD141+ and play a key role in generating immunity against cancer. Thus, cDC1s capture apoptotic tumor cells, migrate to draining lymph nodes, and cross-present tumor antigens to CD8+ T cells [124,125]. Human cDC2s are characterized by the positive expression of CD11c, HLA-DR, CD1c, CD1a, and CD172a, and their role in cancer immunity is less defined [126]. However, cDC2 is associated with a good prognosis in many human cancers. Binnewies showed the crucial role of cDC2s in supporting the antitumor effect of CD4+ T cells and response to anti-PD-1 therapy [127].

In several malignancies, a high density of tumor-infiltrating cDC1s has been linked to a better prognosis. Broz et al., were among the first to show that CD103+ cDC1 significantly activates tumor-specific CD8+ T cell responses within the TME. They identified a DC gene signature based on the ratio of CD103^+^/CD103^−^, associated genes that provide a strong pro-immune survival signal in different cancer, including head-neck squamous cell carcinoma, suggesting that CD103+ cDC1s are critical for robust tumor control in both mice and humans [128].

DCs are not present inside the brain parenchyma in homeostatic conditions, but they can enter the brain tissue via afferent lymphatic vessels or endothelial venules in pathological conditions, such as cancer [129].

The cDC1s are fundamental in antitumor immunity and immunotherapy effectiveness [82,128,130,131,132,133].

Still, the specific role of cDCs in GBM is yet unknown. In the context of cancer, mature DCs can act in two manners: immunostimulatory or immunosuppressive. DCs can, in fact, stimulate the immune system to attack tumor cells. In particular, DCs stimulate both the cell-mediated and humoral anti-tumor T-cells activation [134,135], and tumor cells respond by generating immunosuppressive molecules that alter the composition of the TME, delaying DCs activation and migration, and finally evading immune regulation [136]. To stimulate a T effector cell response against cancer cells in brain tumors, DCs may recognize and deliver tumor-derived antigens inside the brain tissue [137,138].

Currently, research indicated that cDC1s subpopulations with an amplified phagocytic capability were present inside the brain tumor microenvironment [139,140].

In glioma, an increase in T-cells, neutrophils, and pDCs number were associated negatively with TAM/monocyte incidences, while T-cell abundance positively correlated with pDCs and cDCs incidences [140]. Tumors are known to impact DC infiltration, differentiation, recruitment, survival, and functions through various approaches [141]. The functioning of cDCs in cancer was also influenced by tumor-derived substances and metabolites generated by other immune cells. The negative inhibition of cDC1s’ capacity to cross-present tumor antigens has been linked to the accumulation of MDSC-derived oxidized lipids in the cells [82,133].

In GBM, tumor cells generated fibrinogen-like protein 2 (FGL2), which interfered with GM-CSF signaling, inhibiting the activation of CD103+ cDC1s, decreasing the CD8+ T-cell response, and allowing the tumor to escape immune surveillance [142]. Prostaglandin E2 (PGE2) from glioma cells was observed to boost the production of IL-10 by DCs, resulting in the development of a regulatory response in CD4 T cells and a reduction in effector lymphocyte stimulation [143]. Some research has focused on investigating the roles of Nrf2, a redox-sensitive transcription factor, in glioma DCs cells and their impact on the downstream T cell proliferation and cytotoxicity of glioma cells. The TME of GBM causes an increase in Nrf expression in DCs, resulting in the inhibition of DCs maturation and, as a result, a reduction in effector T-cell activation. Inhibition of Nrf2 pathways improves CD80+ and CD86+ DCs maturation in glioma-conditioned media and partially restores the production of bioactive cytokines [34,144].

Wang J. et al., found that inhibiting the redox-sensitive transcription factor Nrf2, in DCs in glioma-exposed milieu increased DC maturation, T-cells activation, and glioma cells cytotoxicity. As a result, DCs exposed to GBM antigens boost Nrf2 expression, which is linked to an immunosuppressive condition, higher IL-10 expression, and decreased CD80, CD86, and IL12 expression [3,144].

A distinct repository of transcription factors, including interferon regulatory factor 8 and 4, and E2-2, regulates the development of each DC subpopulation. cDC1 cells, which are IRF8-dependent, and express CD141 and CCR7, move from the tumor site to lymph nodes, activating CD8+ T anti-tumor cells. The existence of cDC1 cells in the TME is linked to immune-mediated rejection and might be a useful target for DC-based cancer treatments [125,145,146].

The arrangement of DCs in the CNS has not been thoroughly defined, and many questions about their role in GBM’s TME remain unanswered. Flores et al., demonstrated that HSC CCR2+ cells move from the bone marrow to intracranial PD-L1 resistant brain tumors (GBM and medulloblastoma), where they produce APCs like Mo-DCs that activate the CD8+ T-response against tumor cells. These findings might open the door for DC therapy as a treatment option for cancers resistant to checkpoint blockage [147].

Some in vivo studies have shown that using TLR3 and Flt3L increases DC activation and improves anti-tumor response when combined with checkpoint blockade treatment [148,149].

Based on the data mentioned above, the purpose of DCs-based therapeutics is to increase cDC1 in amplifying tumor identification and destroying tumor cells by the immune system, discoveries presented below in the Chapter Immunotherapy.

### 2.2. Tumor Infiltrating Lymphocytes (TILs)—T Cells: CD8+ Cytotoxic T Cells, CD4+ Helper T (Th) Cells, and Regulatory T Cells (Tregs)

At first sight, the presence of immune effector cells in the tumor mass indicates a successful immune response. Nonetheless, the majority of TME immune cells are functionally impaired in some manner, with immune cell populations being changed into phenotypes that aggravate immune system dysfunction. Tumor-infiltrating lymphocytes (TILs) are represented by CD4^+^ T helper (Th), CD^8+^ T cytotoxic (Tc), and regulatory CD4^+^CD25^+^FoxP3^+^ T-cells (Tregs) [150,151].

#### 2.2.1. Tumor-Infiltrating CD8+ T Cells

CD8+ cells have been revealed to play a decisive function in tumor progression control. Nevertheless, multiple parameters, especially the presence of dendritic cells as principal orchestrators of anticancer immune responses, are required for the recruitment and activation of these immune cells at the tumor site [152].

CD8+ cytotoxic T cells are cellular immune effectors that are critical for tumor cells death, but they are unusual in the GBM parenchyma, representing less than a fourth of all CD3+ T cells. In vitro, compared to healthy control cells, T cells isolated from GBM patients are less sensitive to direct anti-CD3 activation, indicating an immunosuppressed state [153].

Longer survival was linked to increased CD3+ T-cell infiltration in GBM, independently of age, MGMT promoter methylation, or post-operative therapy. Within TME, a decrease of systemic CD4+ T-cells numbers as well as an over-expression of inhibitory receptors, such as CTLA-4, CD73, and CD39 was observed, resulting in a decrease in T-cells activity [150]. Lohr J. et al., showed that infiltration of T-cell subsets increased from low-grade to high-grade tumors, and within primary GBM, high numbers of intratumoral T cells (cytotoxic and helper) significantly correlated with better survival [154].

GBM progression causes multiple immune dysfunctions locally and systemically. In consequence, it was proved that GBM has an individually T-cell signature, respectively the enhanced expression of many co-inhibitory immune checkpoints, as well as the diminished functional capacity of glioma-infiltrating T cells. Different signatures were also seen among infiltrating T cells, independent of where these tumors were injected into the body, suggesting that diverse tumors may generate different exhaustion patterns [151].

CD8^+^ T cells will mature into cytotoxic effector cells that generate IFNγ, granzyme B, and perforin. CD8^+^ T lymphocytes selected from GBM tissue had the phenotype CD8^+^CD25^−^, indicating that they were not activated [155].

#### 2.2.2. CD4+ Helper T (Th) Cells

In glioma, the percentages of CD4+ tumor-infiltrating T lymphocytes rose as tumor grade increased, respectively 39% in WHO grade II, 73% in WHO grade III, and 98% in grade IV. On the other hand, CD8+ population was found in the majority of the glioma patient specimens regardless of grade, but less abundant than CD4^+^ T lymphocytes [156].

Lohr J. et al., demonstrated that infiltrating T-cells were regularly identified in WHO IV fibrinogen-positive GBM zones, supporting the idea that leaky arteries prevalent in GBMs, encourage T-cell transmigration. Increased CD3^+^Foxp3^−^ T cell infiltration was linked to the positive expression of ICAM-1 on vascular endothelial cells. T-cell transmigration is mediated directly by CAM molecules expressed on GBM endothelial cells and is blocked specifically by TGF-signaling activity inside GBM endothelial cells [154]. The lack of appropriate T-cell activation in TME is because antitumor T-cell responses are repressed by cytokines secreted by glioma cells, such as TGFβ and IL-10 [157].

Furthermore, glioma-related B7-homologue 1 (B7-H1) was identified as a strong inhibitor for activating CD4^+^ and CD8^+^ T-cell, evaluated by the increase of cytokine production, such as IFN-γ, IL-2, IL-10, and the expression of CD69, the T-cell activation marker. IFN-γ significantly increased B7-H1 expression and thus could have a major impact on the effect of T-cell tumor cell interactions. It could be a new way for glioma cells to escape from immune detection [158].

Within the tumors, a robust correlation between CD4^+^ effector memory T-cells and MDSCs CD14^−^CD15^+^ was identified. Compared to their blood-derived counterparts, tumor-derived CD4+ effector memory T-cells showed high levels of PD-1 and were functionally fatigued. On tumor-derived MDSCs, there was a considerable up-regulation of PD-L1, and T-cell co-culture tests revealed that glioma-infiltrating MDSCs might increase PD-1 expression on CD4+ effector memory T-cells [24,88].

#### 2.2.3. Regulatory T Cells (Tregs)

Regulatory T-cells (Tregs), known as immunosuppressive T-cells, negatively modulate the immune response and protect from autoimmune disease and are the main mediators of immunological tolerance [159,160,161]. Tregs have an important impact on tumor development, immunological microenvironment, and patient prognosis. In the CNS were identified two types of Tregs: thymus-derived natural Tregs (nTregs), which account for the majority of brain-tumor resident Tregs, and induced Tregs (iTregs), which develop from mature CD4+ conventional T cells outside of the thymus [162,163,164].

The processes by which Tregs are recruited in many cancers, including GBM, have yet to be discovered. The presence of Tregs, even in GBM parenchyma, is linked to GBM immune-escape potential, and their number is associated with glioma grade [156,160,161].

GBM tumor cells assist the recruitment and maintenance of Tregs in the TME by secreting soluble molecules such as CCL22 [165], and the number of Tregs has been shown to have an inverse rapport with patient survival [166]. Treg ablation eliminates T-cell proliferative aberrations, allowing in vitro T lymphocytes from GBM patients to function at levels equivalent to healthy controls. As a result, eliminating Tregs might reestablish tumor immune evasion, making tumor immunotherapy or conventional treatment more effective [167].

Some research has shown that numerous CD4+ cells are represented by Tregs, which suppress the IFN-based antitumor response due to the production of Th-2 polarizing cytokines, including IL-10 and TGF [159,167]. TGF-1 and TGF-2 suppress T-cell infiltration in the CNS via inhibiting ICAM-1 and VCAM-1 expression; this data suggests that anti-TGFs treatments might improve immunotherapy efficiency [154]. Tregs are recruited through the binding of CCR4 on Tregs surface and CCL22 released by GBM tumor cells and CD163+ TAMs, induced by tumor-derived CCL20 and osteoprotegerin [168,169]. The expression of the tumor-enzyme indoleamine 2,3 dioxygenase (IDO), linked to tumor growth and worse survival rates, is another factor in Treg chemotaxis [170,171]. In combination with standard chemotherapy, mAb directed against IL-2R (CD25) and CTLA-4, cytokines/interleukin inhibitors, or glucocorticoid-induced TNFR-related (GITR) agonists, all of which are up-regulated in Treg and activated T-cells, have been investigated. However, only a few patients have shown a positive response to these novel medications [167,172,173].

According to Heimberger A, Treg infiltration is more common in tumors of the astrocytic lineage than in those of the oligodendroglial lineage. Further, a considerable variation in FoxP3 staining according to pathologic type: 83% in gliosarcomas, 53% in anaplastic astrocytomas, 48% in GBMs, and 39% in anaplastic mixed oligoastrocytomas was noted. As the glioma grade grew, so did the number of cells that stained positive for FoxP3. Unfortunately, the number of infiltrating Tregs in GBM patients with unifocal vs. multifocal disease did not vary statistically [156].

Polyinosinic: polycytidylic acid (poly I:C) is an immunostimulator that interacts with toll-like receptor 3 (TLR3) expressed on immune cells. Treatment with checkpoint blockade and poly I:C showed a positive impact on DC activation by decreasing the ratio of tumor infiltrating Tregs. The recruitment, growth, and activation of Tregs are widely established as one of the strategies used by malignancies to develop tolerance. The reduction of Tregs in the tumor can be noticed in the PD-1-treated mice, but without statistical significance; nevertheless, when the combinatorial treatment with poly I:C is used, the difference is substantial compared to the control group. Anti-PD-1 treatment stimulates CD8+ cells to secrete IFN-γ, which supposedly reduces the amount of infiltrating Tregs by inhibiting tumor-induced Treg proliferation and recruitment, without statistical significance [148,174,175,176].

**Figure 2:**
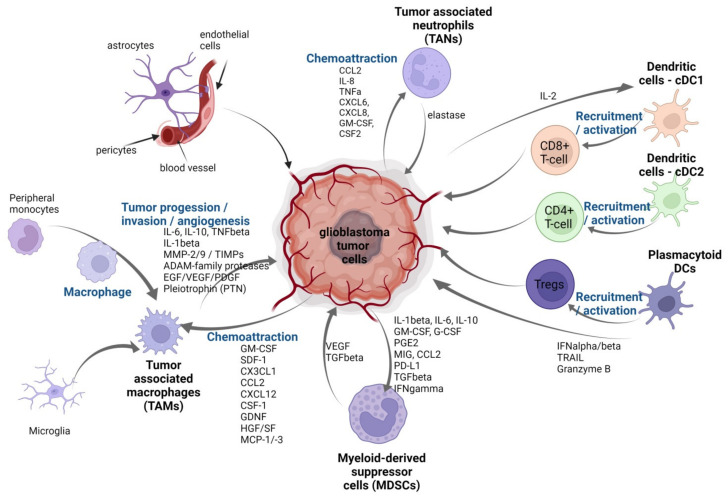
Tumor microenvironment of GBM. The figure depicts immune cells and non-immune cells that have been reported to be associated with tumor cells in TME and their bidirectional crosstalk: tumor-associated macrophages (TAMs—microglia and peripheral monocytes), dendritic cells: cDC1 cDC2 , plasmacytoid DCs, myeloid-derived suppressor cells (MDSCs), tumor-associated neutrophils (TANs). Illustration created with Bioreder.com.

To summarize, the interactions between immune and other cells in the tumor microenvironment are bidirectional and intricate, requiring reprogrammed control, and thus creating a new niche to support cancer cell survival and development (Figure 2).

## 3. Dysregulated Inflammatory Cascade of Chemokines and Their Specific Receptors Promotes Tumor Development

Multiple mediators and regulators, including cytokines, chemokines, angiogenic and growth factors, prostaglandins (PGE2), reactive oxygen species, and so on, are generated by different cell types in the GBM microenvironment, resulting in a complex linkage between tumoral and immunological compartments. Several deregulated chemokines/chemokines specific receptor axes have been implicated in crucial stages of glioma development, resulting in increased glioma malignancy [177].

**Figure 3:**
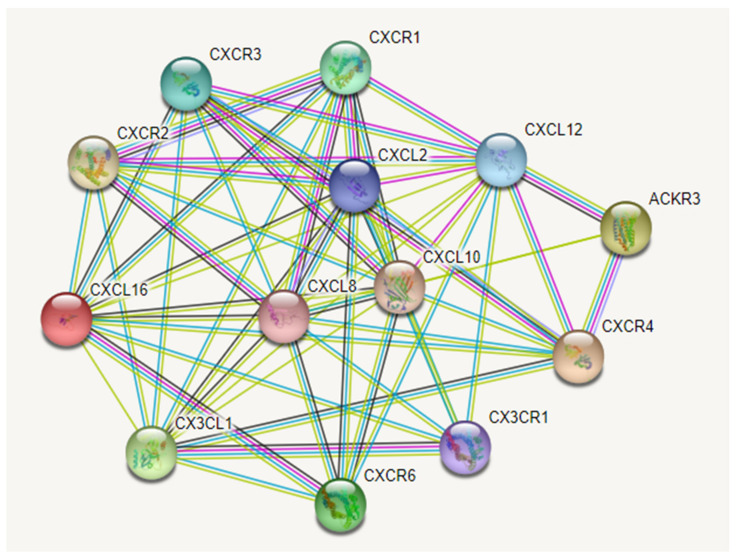
The most important chemokines (CXCL) and their specific receptors (CXCR) axis involved in glioma development. The colored nodes are represented by query chemokines and the first shell of interactors. Edges represent protein-protein functional associations, assigned with different color codes, as follows: a blue edge indicates known interactions from curated databases, a pink edge indicates known interactions that have been experimentally determined, a green edge indicates predicted interactions in the gene neighborhood, a red edge indicates predicted interactions for gene fusions, a blue-ink edge indicates predicted interactions for gene co-occurrences, a light-green edge indicates other interactions derived from text mining, a black edge indicates coexpression, and light-blue indicates other interactions derived from protein homology. Abbreviations: ACKR3—CXCR7. Image created with String-database (https://string-db.org/, accessed on 5 January 2022).

Chemokines and their specific receptors (Figure 3) can be classified into two distinct classes: proangiogenic and angiostatic. Proangiogenic chemokines are CXCL2, CXCL8, CXCL4, CXCL9, CXCL10, CXCL12, and CCL2, while CXCL9 and CXCL10, when combined with CXCL4, can inhibit angiogenesis [178]. CXCR2 is mostly responsible for proangiogenic chemokine activity [179], while CXCR3 is responsible for angiostatic actions [178].

### 3.1. CXCL2/CXCL8 and Their Receptor CXCR2

Recently, besides VEGF, an alternative angiogenic signaling pathway via CXCL2/CXCL8 and its receptor CXCR2 was identified as an essential part of angiogenesis in glioblastoma [180].

In T98G, Hs683, and U373 glioma cells, the expression and secretion of CXCL2, CXCL3, and CXCL8 were measured after various temozolomide (TMZ) treatments. As a result, it was noted that the astroglioma cells treated with temozolomide for a long time exhibit in vivo a certain level of resistance to treatment, which was linked to CXCL2 up-regulation (to a lesser extent, CXCL3, and CXCL8). Furthermore, the transient down-regulation of CXCL2 in oligodendroglioma cells significantly reduced their proliferation rate, and thus CXCL2 directly impacts glioma cell biology [181].

Yang L. et al.’s results revealed that the high expression of CXCR2 was correlated to the degree of malignancy and recurrence of the tumor. Also, an in vitro experiment, performed on a glioblastoma cell line, revealed that cellular migration was reduced when the CXCR2-specific inhibitor SB225002 was applied [182].

Brandenburg S. et al., noted that in an in vitro experiment, CXCL2 was strongly up-regulated and showed better angiogenic activity than VEGF. Treatment with a CXCR2-antibody during initial tumor growth of the CXCL2-CXCR2 signaling pathway revealed significantly diminished volumes of glioma in transgenic mice [183]. Considering all the above mentioned, the CXCL2/CXCL8 signaling through CXCR2 represents an essential signaling pathway that needs to be explored in GBM.

### 3.2. CXCL8 and His Receptors CXCR1 and CXCR2

Apart from CXCL2, the more well-known chemokine CXCL8 (IL8) is also a CXCR2 ligand linked to a pro-angiogenic action in human malignancies [184,185].

The significance of up-regulated interleukin-8 (IL-8/CXCL8) and its receptors (*CXCR1, CXCR2*) levels in glioma was explored by many researcher groups. They demonstrated that CXCL8 is expressed and secreted at high levels in vitro and in vivo, and new research support it as essential to neovascularization and progression of GBM. Secretion and positive cellular expression for CXCL8 and its specific receptors were exhibited in glioma cells, neurons, neutrophils, macrophages, lymphocytes, endothelial cells, and glial cells; thus, each of these cell types may contribute to gliomagenesis [178].

The proangiogenic activity of CXCL8 occurs predominantly following binding to CXCR2, activity highlighted by the following events through endothelial cell response: the enhancement of endothelial cell proliferation, chemotaxis, survival, and protease activation [186]. Pro-inflammatory cytokines (IL-6), anti-apoptotic proteins from the Bcl-2 family (Bcl-xL), or proteolytic enzymes (MMP-2 and -9) determine the increase of CXCL8-CXCR1/2 protein expression and its promoter activity in glioblastoma cells. The activity of CXCL8-CXCR1/2 is related to glioma progression, epithelial-mesenchymal transition, vascular mimicry, inflammation, tumor angiogenesis, and recurrence [178]. Up-regulation of CXCL8 in glioblastoma cells was also shown to be caused by Bcl-xL and is mediated through a nuclear factor-kappa B (NF-kB)-dependent mechanism [187]. An increased level of anti-apoptotic proteins, mainly Bcl-2 and Bcl-xL, and a decreased level of pro-apoptotic proteins Bax and Bcl-xS determined the increased tumor cell survival mediated by CXCL8. Additionally, higher CXCL8 expression was associated with increased production of proteolytic enzymes, particularly MMP-2 and -9, which also contribute to the enhanced glioma cell invasion [188].

CXCL8 mRNA and protein levels were shown to be higher in grades II, III, and IV astrocytomas and anaplastic oligodendroglioma, indicating that CXCL8 expression is linked to glioma grade [186].

CXCL8 expression levels were observed to be strongly related to progression and poor prognosis in human glioma, according to Chen Z. et al., According to his research, CXCL8 accelerated the epithelial-mesenchymal transition in glioma cells via activating the JAK/STAT1/HIF-1/Snail signaling pathway [189].

According to Samaras V et al., IL-6 and IL-8 expression in GBM samples were positively linked with histological grade, indicating they may play a role in stimulating neoplastic glial cell development via an autocrine or paracrine mechanism. Moreover, the correlations of pro-inflammatory cytokines, such as IL-6 and IL-8 with angiogenic factor VEGF and COX-2 expression levels, as well as the coordinated expression and structural relationship of these molecules expressions, confirm the close collaboration of these molecules in terms of tumor-induced angiogenesis. This association could be subordinate to the stimulation of multiple signaling pathways that influence the process of vascular formation [190].

Measured in cerebrospinal fluid (CSF) or serum from astrocytic vs. non-tumoral patients, CXCL-8 level was elevated in cerebrospinal fluid, and CCL2 level was decreased in serum, which may be helpful in the management of CNS astrocytic brain tumors [191]. Also, CXCL8 protein expression was present in the cyst fluid of low-grade astrocytomas, anaplastic astrocytoma, glioblastomas, and oligodendroglioma grade III [192].

Angara K. et al., demonstrated an up-regulation of CXCL8-CXCR2 pathway, in high-grade gliomas tumors that are resistant to antiangiogenic therapies through another mechanism of neovascularization called vascular mimicry [193].

CXCL8 mRNA levels were found to be elevated in glioblastomas and anaplastic astrocytomas compared with other gliomas; moderately correlated with macrophage counts, which suggest that either CXCL8 might be produced by activated macrophages or IL-8 production by gliomas might have a significant role in attracting and activating macrophages [194].

### 3.3. CXCL10/IP-10 and His Receptor CXCR3

CXCL10 chemokine binds to the CXCR3 receptor and exerts different functions on carcinogenesis depending on the spliced variant: whereas CXCR3-A promotes cellular proliferation, CXCR-B exerts apoptosis and growth inhibition [195].

Maru S.V. et al., analyzed the expression of chemokine receptors and chemokine production in glioma cells. They showed an increased production of CXCL10, which induced an ERK1/2-dependent increase and DNA synthesis, suggesting that expression of CXCL10/CXCR3 axis have an important role in the proliferation of grade III astrocytoma and grade IV glioblastoma cells [196].

Furthermore, Zhu H. et al., discovered increased recruitment of CD68 positive cells, an immune cells marker, to the surgical site. When comparing debulking +anti-CD47 tumors to non-debulking +IgG cancers, protein analysis revealed an increase in CXCL10 and a decrease in angiogenic protein expression. Results recommended a combination between surgical resection and anti-CD47 blocking immunotherapy, which promotes inflammatory response and prolongs survival [197].

Using RT-PCR, it was found that CXCL10 mRNA was expressed in GL261 glioma cells. Additionally, in situ hybridization investigation identified CXCL9 and CXCL10 mRNAs in tumors from CXCR3-deficient mice, demonstrating that CXCL9 and CXCL10 expression were unaffected by this impairment. CXCL10 is expressed in murine glioma GL261 cells in vitro, while CXCL9 and CXCL10 are expressed in vivo in GL261 tumors. This study mentions a variation of CXCL10 expression in different human glioma lines [198,199].

In a recent study, Shono K. et al., showed that celecoxib, a new class of nonsteroidal anti-inflammatory drugs, suppressed the CXCL10/CXCR3 and CCL2/CCR2 axes, which may have antitumoral effects in a mouse malignant glioma model, in addition to intrinsic and extrinsic apoptotic effects induced in GSCs. In the glioma mouse model, celecoxib treatment decreased protein expression and mRNA levels of Ccl2, CxcL10, and Cxcr3, both in the tumoral and peri-tumoral tissue. CXCL10 and CCL2 were also found in the tumor and TME, where they co-localized with Nestin+, Iba-1+, CD163+, or CD16+ cells, suggesting that they had a similar distribution and location. Celecoxib therapy, on the other hand, lowered Ccl2 and Cxcr3 protein and mRNA levels in GSCs, which were associated with NF-κB signaling suppression and Ccl2 silencing by siRNA suppressed cell growth [200].

### 3.4. CXCL12 and His Receptors CXCR4 and CXCR7

The CXCL12—CXCR4 axis is extensively expressed in the normal brain and plays an important role in CNS development, but its participation in glioblastoma may be an example of tumor cells “hijacking” physiological processes in the CNS [201].

The activity of CXCL12-CXCR4 is related to glioma progression, cancer cell-tumor microenvironment interaction, cellular invasion, and tumor angiogenesis. Angiogenesis is one of the key hallmarks of GBM, and CXCL12 binding to CXCR4 participates in this process via boosting VEGF release, the most important angiogenic stimulant [202].

In addition, through PI3K/AKT signaling, CXCL12 and its specific receptor CXCR4 have been shown to increase VEGF synthesis mediated by GSCs and tumor angiogenesis [203].

Hypoxia influences the CXCL12-CXCR4 axis, which results in a positive loop: CXCL12 stimulates the synthesis of VEGF, which in turn boosts CXCR4 expression [204]. Hypoxia is another hallmark in GBM and causes an increase in HIF-1 expression, which boosts CXCL12 production in tumor cells, influences tumor cell spreading via CXCR4 receptor binding to endothelial cells, and stimulates the VEGF expression, resulting in enhanced angiogenesis. Thus, CXCL12 appears to cause vasculogenesis in GBM in addition to angiogenesis [205]. All these activities increased the synthesis of many chemokines, including CXCL8, chemokines controlled by the MAPK pathway [206].

When the expression alterations in initial GBM diagnosis and recurrence after treatment were evaluated, VEGF-VEGFR2/VEGFR1 axis, HIF1α, urokinase plasminogen activator (uPA), CXCL12, and CXCR4, changes were shown between RNA and protein expression. CXCR4 and CXCL12 expressions were found to be raised after GBM recurrence, while HIF1a and VEGFR2 expressions were found to be decreased. After chemoradiation, recurrence of GBM is linked to a transition in angiogenic pattern from VEGFR2-HIF1 to CXCL12-CXCR4 [207]. Based on these observations, glioblastoma recurrence following radio-chemotherapy is linked to an angiogenic transition in the pattern of chemokine production to the proangiogenic and protumoral CXCL12-CXCR4 pathway.

CXCR4+ cells are directly involved in angiogenesis by binding CXCL12 to its receptor on endothelial cells and attracting endothelial progenitor cells [208]. Additionally, CXCL12 may indirectly stimulate angiogenesis by inducing secretion of pro-angiogenic VEGF, CXCL8, and CXCL1 by endothelial cells and leukocytes expressing CXCR4 [209].

According to many studies of glioblastomas and neuroblastomas, CXCR4+ monocytes attracted to tumors induce the formation of new blood vessels within the tumors. Thus, monocytes initially establish themselves within the tumor’s perivascular regions, then release pro-angiogenic factors, followed by recruitment of bone marrow-derived endothelial and perivascular progenitors, and finally form the new blood vessels [210,211].

CXCR7 is an alternative receptor with increased affinity for CXCL12, which complicated the understanding of the CXCL12/CXCR4 axis. While CXCR4 and CXCR7 receptors are moderately expressed on normal endothelial cells, CXCR7 expression on these cells inside of the TME is significantly increased, and it has recently been proposed as a biomarker of tumor vasculature in cancers, like renal carcinoma and gliomas [212].

Nf1+/TAMs generate paracrine factors and chemokines capable of enhancing Nf1-deficient astroglial cell proliferation [213], such as stroma-derived factor-1 (SDF-1/CXCL12), which was found to be increased in Nf1+/− TAM relative to wild-type microglia [214,215]. SDF-1 enhances optic glioma cell survival via the CXCR4 receptor, whereas blocking CXCR4 inhibits tumor development in vivo. Using RNA-sequencing technologies, a more comprehensive characterization of TAMs that promote tumor maintenance has been carried out [216,217]. CXCL12 is a powerful microglia and macrophage recruiting molecule that is particularly effective in attracting TAMs to hypoxic regions [218].

### 3.5. CXCL16 and His Receptor CXCR6

The chemokine CXCL16 has a unique structure that distinguishes it from other human chemokines [219]. Soluble and transmembrane forms have different activities in cell recruitment. The transmembrane variant generates strong cell-to-cell adhesion by interacting with CXCR6, but soluble CXCL16 functions as a chemoattractant, generating migratory signals [220].

CXCL16 is highly expressed in human gliomas, and compared with the normal brain were restricted to brain vascular endothelial cells. In experimental models, in vitro and in vivo, mRNA and protein expression of CXCL16 is up-regulated by TNFalpha and IFNgamma [221].

GBM cells secreted CXCL16, a chemokine that regulates tumoral development, invasion, angiogenesis, and the maintenance of an immunosuppressed TME [222,223].

An over-expression of CXCL16 was noted in glial tumor and stroma cells, while CXCR6 expression is more likely associated with glioma-stem cells [224].

CXCL16 is generated by tumor cells and supports the anti-inflammatory/pro-tumor polarization of glioma-associated microglia/macrophages, as well as inhibiting microglia polarization toward an inflammatory phenotype in response to LPS and IFN stimulation, according to recent research. Besides, the CXCL16/CXCR6 pathway promotes tumor development and invasion, in different cell lines, such as mouse and human GBM cells. In light of these findings, the CXCL16/CXCR6 pathway might be a noble target for microglia phenotype modulation to decrease inflammation and reduce tumoral growth [225].

CXCL16 induced high expression of CXCR6 on glial precursor cells. Stimulation by CXCL16 activates the PI3k/Akt pathway and afterward the transcription factor AP-1. As a result, glial precursor cells multiply and move to the sites of CXCL16 production. Hence, CXCL16–CXCR6 axis promotes malignant transformations and contributes to human GBM cell growth, invasion, and migration [220].

Hattermann K. et al., found that microglial, endothelial, and tumoral cells express, in situ and in vitro, the transmembrane chemokine CXCL16. In glioblastomas, CXCR6 is restricted to a subset of proliferating cells that are Musashi+, Nanog+, Sox2+, and Oct4+. Also, CXCR6+ cells made for about 90% of Musashi+ cells. As a final point, CXCL16 was demonstrated to be considerably expressed by glial tumor and stroma cells, whereas CXCR6 was found in stem cell fenotype cells [224].

### 3.6. CX3CL1 and His Receptor CX3CR1

Erreni M. et al., demonstrate that CX3CR1 mRNA and protein levels were equally expressed in low- and high-grade tumors, whereas CX3CL1 (fractalkine) was found in oligodendrogliomas, anaplastic astrocytomas, and glioblastomas. The CX3CL1 expression was inversely correlated with overall patient survival, and this increased expression of grades III-IV gliomas suggests that this axis is involved in the tumor’s malignant behavior [226].

CX3CR1 is commonly expressed by microglial cells in the normal brain, where it has been established as a reliable marker for in vivo microglia. CX3CR1 loss impacts synaptic plasticity throughout development since the CX3CL1-CX3CR1 signaling pathway is crucial for neuron-microglia contact [227]. Nevertheless, there is still controversy over the role of CX3CL1 in tumor-directed TAM migration [16,228,229].

CX3CL1 and CX3CR1 have a positive immunological expression on tumor cell membranes, and soluble CX3CL1 accumulates in cell culture supernatants, indicating that gliomas produce this chemokine on a regular basis. The use of a neutralizing mAb to block endogenous CX3CL1 function dramatically slowed tumor cell aggregation and boosted glioma invasiveness. Additionally, TGF-beta1 therapy reduced CX3CL1 mRNA and protein expression, which is a major regulator of glioma cell invasiveness [230].

In the light of these findings, the up-regulation of the above-mentioned chemokine-specific receptors axes are beneficial for gliomagenesis because they are involved in glioma onset, proliferation, growth enhancement, angiogenesis, aggressiveness, and influence various pathophysiological mechanisms.

## 4. Cell Therapy—A Novel Avenue to Pursue in GBM

Pharmacological therapies of GBM, including monoclonal antibodies or tyrosine kinase inhibitors, failed to provide sustained and effective results in GBM therapy, improvements in outcome have been/being modest [3]. Therefore, the focus shifted increasingly towards cellular therapies—therapies using autologous immune cells to destroy cancer cells. However, despite the number of immune cells, cancer cells are subjected to a high level of immune evasion while inducing immunosuppression, which poses a considerable hurdle for their immune-mediated clearance. An in-depth examination of GBM’s microenvironment, on the other hand, may lead to innovative treatment chances to enhance patients’ outcomes on novel active and passive immunotherapies such as immunization, gene therapy, checkpoint inhibition, and adoptive T-cell therapies [3].

### 4.1. CAR-T Immunotherapy

CAR-T immunotherapy is a tumor-targeted therapy in which a receptor is transduced into T cells to create a chimeric cell—CAR-T cells, which are then multiplied to enormous numbers in vitro and injected back into the patient, causing them to specifically identify antigens using the transfected receptor and killing the tumors [231,232].

CAR-T immunotherapy can pass the blood-brain barrier, permitting it to target tumor cells that are difficult to reach surgically [233,234].

Since CAR-T cell therapy opened the door to a new era of cancer treatment, many CARs have been created to target glioma, including CARs targeting IL13R2, EGFRvIII, HER2, and CD70 [232].

Brown et al., presented the case of a patient with recurrent GBM who was treated with CAR-T cells and discovered that IL13R-2 was a promising immunotherapeutic target in GBM. Over the course of 220 days, several CAR T cell infusions were given via two intracranial delivery routes: infusions into the excised tumor cavity followed by infusions into the ventricular system. Intracranial infusions of CAR13 cells targeted by IL13R2 have not been linked to any toxic effects of grade 3 or above. Regression of all intracranial and spinal tumors was found after treatment with CAR T cells, along with corresponding increases in cytokines and immune cells in the cerebrospinal fluid [235].

A CD137 costimulatory domain has been added to second-generation IL13R2 CAR-T cells, and an artificial platform has been employed to enrich central memory T cells. In glioblastoma animal models, the antitumor efficacy and stability of these CAR-T cells are higher than those of first-generation IL13R2 CAR-T cells [236].

Infusion of HER2 CAR-T cells causes T cell proliferation and IFN- and IL-2 release in an orthotopic murine glioblastoma xenograft model, mediating the regression of HER2-positive glioblastoma [237].

In a phase I clinical trial, the safety and feasibility of HER2 CAR-T cells were evaluated. The findings revealed that the HER2 CAR-T cells were well tolerated and provided therapeutic benefit in glioblastoma patients [238].

Another research provides GD2 as a target for CAR-T cell therapy in human glioblastoma in vitro and in vivo preclinical models targeting primary cancer cells. It is the first to explore autologous setting vs. GD2 as a target. GD2 has been identified as a possible target for CAR-T treatment for brain cancer by several research groups [239,240,241,242].

Recently, CAR-T therapy has been used to treat solid tumors, but many problems remain unsolved, mainly regarding CAR-T cells that enter the TME of solid tumors such as G BM, maintaining their viability and ability to identify tumor cells quickly and precisely and overcoming immunosuppression.

### 4.2. Role of NK Cells in the Treatment of GBM

Natural killer (NK) cells are lymphoid cells that serve as the first line of defense against infection and antitumor immunity. Their inhibitory surface receptors recognize MHC class I molecules on the surface of normal somatic cells. Current research focuses on imitating NK cell activity to reproduce their attacking and immune-killing effects, as NK cells are relentless in their targeting of tumor cells and difficult to escape [243,244].

The current development of NK cell-based therapy can be divided into 3 directions: NK cells are used directly to kill GBM cells; Immune cell therapy treatments that include NK cells plus immune checkpoint inhibitors or drugs that target immune-related genes; or particular antibodies that target proteins that protect NK cells from immunosuppression, and CAR-NK (chimeric antigen-modified natural killer) cell therapy [245].

GBM systemic metastases are prevented by NK cells. GBM mortality can be directly produced by transplanting NK cells into a GBM model, but the challenge of this strategy comes in the ambiguity of the NK cell transplantation procedure. GBM is thought to be caused by CMV infection interfering with the immunological response of NK cells [246,247].

Yvon et al., used NK cells generated from umbilical cord blood in an immunotherapy approach for GBM, although this method is restricted by immunosuppressive cytokines in the tumor microenvironment [248].

Moreover, many researchers have suggested NK cell-related combination immunotherapies. In two preclinical tumor models, it was shown that adding the immune checkpoint inhibitor PD-1 with a combination antibody could drive important infiltration of NK cells and T cells while also inhibiting tumor development [249].

None of these combination immunotherapies have been investigated in terms of the particular mechanism of action of other cells other than NK cells in helping to eliminate GBM. Systemic metastasis of GBM is uncommon due to NK cells’ inherent immunity, and researchers are investigating combining radiation, immune cells, and immune checkpoint inhibitors to improve GBM treatment. Although a vast amount of experimental data shows that the aforementioned strategies can improve immune cell infiltration, there are still many issues with the practical use of this process. The FDA approved CYNK-001, a natural killer cell therapy, as an experimental novel medication for treating glioblastoma multiforme. CYNK-001 is a cryopreserved allogeneic NK cell treatment produced from placental hematopoietic stem cells for intravenous or intratumoral administration. This was the first clinical trial of an allogeneic NK cell treatment for glioblastoma multiforme in the United States [250].

As a result, using NK cells in the brain to kill GBM still has many challenges, the most important and difficult of which is preventing NK cell cytotoxicity from being inhibited. In the GBM tumor microenvironment, NK cells are functionally suppressed.

Another treatment option for GBM is CAR-NK therapy, which has recently been translated in clinical studies.

One group of researchers discovered that ErbB2 protein expression was overexpressed in a substantial proportion of GBM samples and employed ErbB2/HER2-specific NK cells to target GBM, proposing “CAR-NK” cells—human NK cells that express ErbB2-specific chimeric antigen receptors [251].

The therapeutic benefits of NK-92/5.28 cells on endogenous antitumor immunity were also demonstrated in vitro and in vivo on GBM cell culture and orthotopic GBM xenograft models, as well as the therapeutic effects of these CAR-NK cells on endogenous antitumor immunity. Murakami et al., also provide a strategy for targeting epidermal growth factor receptor variant III (EGFR vIII) and inducing antitumor activity in GBM cells using a new NK cell line containing a chimeric immune antigen receptor (CAR-KHYG-1) [252].

Another group showed that CAR-redirected NK cells may efficiently target wt EGFR and EGFRvIII to treat GBM, and that intracranial treatment of NK-92-EGFR-CAR cells can effectively inhibit tumor growth, indicating that this is a promising clinical strategy [253].

Finally, given the huge number of ongoing investigations on targeted NK cell therapy for GBM, this therapeutic approach appears to have a strong possibility of becoming a full-fledged immunotherapy strategy. To sum up, the above-mentioned investigations revealed that clinical trials are needed to assess the safety and efficacy of adoptive immunotherapy using CAR-NK cells.

### 4.3. TAM Therapy

TAMs are macrophages that populate the tumor-surrounding microenvironment to promote tumor growth.

Different therapeutical approaches have been used for targeting TAMs, as follows: (1) TAMs currently present in the TME are removed; (2) monocyte/macrophage recruitment is inhibited; and (3) TAMs are reprogrammed [254].

Depletion of TAMs in different cancer models was obtained using bisphosphonates, respectively clodronate [255] and zoledronate acid [256]. Both are currently undergoing clinical studies.

Another TAM-targeting approach is to inhibit circulating monocytes recruitment by blocking the important chemokine signals of CCL2/CCR2 [257] and CXCL12/CXCR4 [258,259] axis. Thomas R.P. et al., noted that in newly diagnosed GBM patients treated with Plerixafor—a CXCR4 inhibitor, this was well tolerated in addition to Bevacizumab and improved local control of tumor recurrences [258].

The third TAM-targeting approach was based on restoring the antitumor properties to TAMs [260], targeting the SIRPα-CD47 pathway, toll-like receptor (TLRs: TLR 7/8/9), CSF1/CSF1R pathway, CD40, PI3K, and NF-kB [261]. SIRP identifies CD47, which acts as a “don’t eat me” signal and is found overexpressed in tumor cells, and is linked to poor patient survival [262]. In GBM, different TLRs ligands agonists were tested as immune adjuvants for immunotherapy [261]. Inhibition of CSF1R causes changes in macrophage polarization and decreases glioma growth [263], and also enhances radiotherapy efficacy and reduces immune suppression in GBM patients [264].

Other investigations have found that macrophages play a direct immune-related function in GBM treatment. Overexpression of Romo1 in bone marrow cells, for example, substantially reduced the immune response within the tumor microenvironment and facilitated glioblastoma progression, showing that overexpression of Romo1 (reactive oxygen species modulator 1) in macrophages may be an essential mechanism of immunological tolerance for glioblastoma [265].

Wnt-induced signaling protein 1 (WISP1) is required to maintain glioma stem cells and tumor-supportive TAMs in GBM, implying that inhibiting Wnt/-catenin-WISP1 signaling could improve GBM treatment and clinical outcome [266].

Macrophage-associated cytokines are utilized to predict the prognosis of GBM patients. Higher levels of IL-6 in the CSF were correlated with a worse prognosis in patients with GBM in both univariate and multivariate analyses. These findings suggest that IL-6 levels are correlated to TAM infiltration and could be a helpful predictive biomarker for GBM patients [267].

Osteopontin (OPN) is a chemokine that attracts macrophages to glioblastoma cells, enhances interplay between tumor cells and the innate immune system, and might be utilized as a therapeutic target [268].

However, these findings have yet to be completely explored. They present innovative perspectives for treating GBM with macrophages, demonstrating that macrophages have diverse functions and will potentially be used in other aspects.

### 4.4. Dendritic Cell Vaccines

Dendritic cells serve as sentinels of the innate immune response when they come into contact with foreign antigens, specifically pathogen-associated molecular patterns [269].

Isolating Dendritic cells from patients, loading the cells with tumor antigens, cultivating the DCs with cytokines to stimulate maturation, and reinjecting the cells back into the body are all steps in creating DC vaccines [270]. Currently, vaccinations are classified as tumor-associated antigens (TAAs), tumor-specific antigens (TSAs), or tumor lysates based on the antigens they contain [271].

TAAs are found everywhere but are produced in larger quantities in tumor cells than in healthy cells, making TAA vaccines easy to make and effective. TAA-based DC vaccines’ clinical application is currently limited due to the following. There are few known TAAs. TAA vaccines may not produce the greatest immune response due to immune tolerance [271]. ICT-107 vaccination resulted in good resistance and a 2.2-month increase in survival in patients with newly diagnosed glioblastoma [272].

TSAs are proteins encoded by mutant genes in tumors, unlike TAAs, which are found in both tumor cells and normal cells. They can be utilized as immunotherapy targets since they are generally fixed in different types of cancer [270]. TSA-based dendritic cell vaccines have the potential to cause a strong targeted inflammatory response against tumor cells while avoiding autoimmune reactions in other tissues [271].

### 4.5. Gene Editing as a Tool for GBM Research and Therapy

The utility of gene editing for GBM knowledge advancement has been solidly argued in human cell lines and organoid models [273], as well as animal models (for details on the genome engineering tools and models used, see [274]). It is continuously used for various purposes, such as screening for therapeutic targets or generation of cellular models and accurate animal disease models. For example, simultaneous editing of targeted tumor suppressor genes *P53, NF1,* and *PTEN* induced early lethal brain tumors, resembling GBM, in mice [275,276].

The most frequently-used gene-editing technology, CRISPR/Cas9, can be used for single target knockout or as a screening tool, using sgRNA libraries, to collect data on genetic regulators of invasion [277], radiation resistance [278], temozolomide resistance [279], interaction to the extracellular matrix [280], apoptosis, or autophagy in GBM (reviewed in [281]). Genome-wide gene editing screens were proven effective for highlighting gene hits outside of core genes and regular pathways described for GBM, such as Wee-1 kinase [282], which prevents mitotic entry, currently targeted by orally available inhibitors and tested in clinical trials [283]. When used in primary glioma stem cells (derived from patients), this tool identified “common GSC fitness genes, despite inherent intratumoral heterogeneity and irrespective of patient tumor genotype” [284].

Although named “gene” editing, CRISPR/Cas system is starting to be used as a tool to target non-coding sequences of the genome, such as micro- and long non-coding RNAs. So far, it has been employed to demonstrate the impact of miR-10b [285] on GBM growth and survival and the role of HOTAIRM1 on high-order chromatin structure in glioma cells [286]. A recent CRISPR interference study identified *lncGRS-1* as a promoter of malignancy in glioma cells [287]. One study employed a nuclease-deficient Cas9 to activate miR-134 and miR-485 expression to induce apoptosis in GBM cells [288].

A major limitation of gene editing attempts is the variability of the outcome. In vitro gene-editing yielded more than anticipated out-of-frame mutations, which may lead to increased effectiveness [282]. However, in vivo, the output of this variability may impact the phenotypic presentation of the disease. Although the same genes are being targeted, frameshift mutations and deletions may vary between similar in vivo models, leading to different pathology types [276], which may impede therapeutic outcomes.

While the limitation of off-target editing activity was achieved in vitro by transient nucleofection [289], in vivo screening methods are just being developed to allow, for example, the identification of glioma suppressors [290]. GBM mouse model delivery of CRISPR/Cas tools has been efficiently achieved only by stereotactic injection, whereas systemic distribution was reported to be less efficient [275]. Currently, other administration pathways, as well as the non-viral delivery of the CRISPR/Cas9 system in mammals, are topics of intense research (reviewed in [291]).

The Cas9 system utility continuously expands toward clinical applications, e.g., to assay *MGMT* methylation and *IDH1/2* mutations in clinical samples [292].

Gene editing is already being used as a powerful tool in immunotherapy studies on GBM. It efficiently knocks out different genes in the already genetically modified CAR-T cells. For example, knocking out PD-1 on CAR-T cells targeting EGFRvIII inhibited the growth of GBM cells in vitro [293] and in vivo [294]. Although not directly related to immunosuppression pathways so far, diacylglycerol kinase inhibition by gene editing was shown to induce resistance of CAR-T cells to soluble immunosuppressive factors using the arachidonic acid signaling pathway and to induce regression of glioblastoma tumor in vivo [295]. Gene editing was also tested to increase CD8+ T lymphocytes’ efficiency in targeting and destroying GBM tumor cells by identifying putative membrane target proteins [296]. Recently, gene editing of natural killer (NK) lymphocytes showed promising results in enhancing cytotoxicity against glioblastoma cells [297,298]. Recently used and approved by FDA as a treatment for multiple refractory myeloma, gene editing of patients’ T-cells will most likely become the next gold standard of care in aggressive cancers, including GBM.

Another novel strategy involves injecting modified viruses into the systemic circulation or directly into the tumor site, causing tumor cells to lyse [299]. As a result, tumoral neoantigens and damage factors disseminate, causing a simultaneous innate and adaptive immune response directed against the tumor [300,301]. Indeed, some pioneering clinical trials showed how oncolytic viruses could revert to “warm” the immunologically “cold” GBM’s microenvironment and increase the response of the tumor to checkpoint inhibitors, as well as to the standard treatments [172,302].

## 5. Conclusions

In this review, we underlined the molecular mechanisms by which GBM cells recruit and modulate other microenvironmental cells. Accumulating evidence has emphasized the critical role of immune cells, like myeloid and lymphoid cells found in the GBM microenvironment, considered cancer progression regulators. Suppressive and pro-tumorigenic myeloid cells that represent the main immune cell population in GBM tumor microenvironment actively confer resistance of GBM to therapy. In addition, dendritic and neutrophils favor tumor cell survival by suppression of the cytotoxic activity of T-cells and generation of NETs, respectively. Despite the advances in GBM research, there is an emerging need for identifying reliable markers that can offer clear information on the active phenotype of these modulator cells and possibly a target to pharmacologically change those phenotypes to support cytotoxic cells. However, many concerns remain unsolved regarding how GBM regulates the landscape of myeloid cells, as well as the processes underlying dynamic heterogeneity in these cells during immunological and therapeutic responses in the setting of GBM.

In recent years numerous studies have been shown that chemokines and their large number of interacting receptors play essential roles in the recruitments of immune cells into the tumor microenvironment, actively promoting the gliomas development and progression, angiogenesis, and tumor metastasis. The multiplicity of chemokine and its receptor interactions could explain the complicated effects of the chemokine system in tumors. Despite significant advances in our understanding of the complex chemokine-receptor system in tumor biology, we still need to dig deeper to understand their prognostic value in GBM, particularly in response to various anticancer therapies.

In the near future, through the use of improved high-throughput technologies, patient-derived GBM organoids and new multiple pharmacological inhibitors, new data on the complex mechanisms underlying the bidirectional interactions between cells of the tumor microenvironment will emerge. Finally, in light of recent progress in genetic editing, the treatment of GBM will probably shift from the current gold standard to cellular therapy, where the pharmacological intervention will support the cytotoxic immune effect.

## Figures and Tables

**Table 1:** Specific surface markers for microglia and bonemarrow-derived macrophages.

Surface Marker	Microglia	BMDMs	References
CD11b	+	+	[18]
CD45	low		
CD68	+	+	
CD206	low		
CCR2	low	+	
CX3CR1	+	low	
SALL-1	high		[61]
TMEM119	high		[62]
SIGLEC-H	+		
P2RY12	+		
SLC2A5	+		
FCRLS	+		
GDA		+	[33]
EMILIN2		+	[33]
HP		+	[63]
SELL		+	[63]

Abbreviations: BMDMs (bone marrow-derived macrophages), SALL1 (Sal-like protein 1), transmembrane protein 119 (TMEM119), SIGLEC-H (Sialic acid-binding Ig-like lectin H), P2RY12 (P2Y purinoceptor 12), SLC2A5 (Solute carrier family 2, facilitated glucose transporter member 5), FCRLS (Fc receptor-like S, scavenger receptor), GDA (Guanine deaminase), EMILIN2 (Elastin Microfibril Interfacer 2), HP (Haptoglobin), and SELL (L-selectin) for macrophages.

## Data Availability

Not applicable.
